# The addition of an amylopectin/chromium complex to branched-chain amino acids enhances muscle protein synthesis in rat skeletal muscle

**DOI:** 10.1186/s12970-020-00355-8

**Published:** 2020-05-27

**Authors:** James R. Komorowski, Sara Perez Ojalvo, Sarah Sylla, Hakki Tastan, Cemal Orhan, Mehmet Tuzcu, Nurhan Sahin, Kazim Sahin

**Affiliations:** 1 Research and Development Department, Nutrition 21 LLC, Purchase, NY 10577 USA; 2 Division of Biology, Faculty of Science, Gazi University, 06100 Ankara, Turkey; 3 Department of Animal Nutrition and Nutritional Disorders, Faculty of Veterinary Medicine, Firat University, 23119 Elazig, Turkey; 4 Division of Biology, Faculty of Science, Firat University, 23119 Elazig, Turkey

**Keywords:** Amylopectin, Chromium, Insulin sensitivity, Muscle protein synthesis, Essential amino acids, Branched-chain amino acids

## Abstract

**Background:**

A previous clinical study reported that the addition of an amylopectin/chromium complex (ACr; Velositol®) to 6 g of whey protein (WP) significantly enhanced muscle protein synthesis (MPS). Branched-chain amino acids (BCAAs) are also well-known to enhance MPS. The aim of this study was to determine if the addition of ACr to BCAAs can enhance MPS and activate expression of the mammalian target of the rapamycin (mTOR) pathway compared to BCAAs and exercise alone in exercise-trained rats.

**Methods:**

Twenty-four male Wistar rats were randomly divided into three groups (*n* = 8 per group): (I) Exercise control, (II) Exercise plus BCAAs (0.465 g/kg BW, a 6 g human equivalent dose (HED)), and (III) Exercise plus BCAAs (0.465 g/kg BW) and ACr (0.155 g/kg BW, a 2 g HED). All animals were trained with treadmill exercise for 10 days. On the day of the single-dose experiment, rats were exercised at 26 m/min for 2 h and then fed, via oral gavage, study product. One hour after the consumption of study product, rats were injected with a bolus dose (250 mg/kg BW, 25 g/L) of phenylalanine labeled with deuterium to measure the fractional rate of protein synthesis (FSR). Ten minutes later, muscle tissue samples were taken to determine MPS measured by FSR and the phosphorylation of proteins involved in the mTOR pathway including mTOR, S6K1, and 4E-BP1.

**Results:**

ACr combined with BCAAs increased MPS by 71% compared to the exercise control group, while BCAAs alone increased MPS by 57% over control (*p* < 0.05). ACr plus BCAAs significantly enhanced phosphorylation of mTOR, S6K1 and 4E-BP1 compared to exercise control rats (*p* < 0.05). The addition of ACr to BCAAs enhanced insulin levels, mTOR and S6K1 phosphorylation compared to BCAAs alone (*p* < 0.05). Serum insulin concentration was positively correlated with the levels of mTOR, (*r* = 0.923), S6K1 (*r* = 0.814) and 4E-BP1 (*r* = 0.953).

**Conclusions:**

In conclusion, the results of this study provide evidence that the addition of ACr to BCAAs significantly enhances exercise-induced MPS, and the phosphorylation of mTOR signaling proteins, compared to BCAAs and exercise alone.

## Introduction

Exercise is known to cause physiological alterations in skeletal muscle, particularly in muscle glycogen reduction and regulation of muscle protein synthesis (MPS) [1, 2]. Several studies have shown that the combination of carbohydrates and protein after exercise replenishes muscle glycogen more efficiently than consumption of carbohydrates alone [3, 4]. Wang et al. [4] reported that post-exercise dextrose and whey protein supplementation increased MPS after exercise compared to placebo, with whey protein probably initiating greater activation of the mammalian target of rapamycin (mTOR) signaling pathway. The branched-chain amino acids (BCAAs) comprised of leucine, isoleucine, and valine are commonly known as the nutrients with the strongest anabolic effect in mammals [5]. Various studies have shown that amino acids, particularly BCAAs and specifically leucine, stimulate MPS in muscle [6–9]. If the body does not supply free amino acids, the protein degradation that occurs after exercise may persist for a longer period of time, thus restricting MPS [10]. During the first hour following exercise, MPS is increased and may continue for 24–48 h, and therefore supplementation with essential amino acids following exercise is vital for MPS [11, 12]. Moreover, studies have shown that consumption of a protein-containing meal directly after exercise promotes MPS in the same manner as complex protein or full-mixed amino acids [13, 14].

Although the anabolic structure of insulin is well described, the role of insulin in the molecular mechanism of MPS in exercised rats is still unclear. Many studies suggest that insulin can play an important role in enhancing net protein balance by reducing protein degradation [10, 15]. In the presence of adequate whole protein and/or essential amino acids (EAAs), insulin stimulates MPS, while at lower blood EAA levels, insulin inhibits protein breakdown [16, 17]. At the molecular level, the phosphoinositide-3-kinase (PI3K/Akt/mammalian mechanistic) target of the rapamycin (mTOR) pathway has a critical role in regulating skeletal muscle protein metabolism and mass [18]. In particular, mTOR facilitates the effects of nutrients and insulin on protein synthesis. Activated mTOR promotes mRNA translation initiation and protein synthesis by phosphorylating eukaryotic translation initiation factor 4E (eIF4E)-binding protein 1 (4E-BP1). This supports the formation of eIF4E from the inhibitor eIF4E•4E-BP1 complex and the mRNA translation initiation formation, promoting the eIF4E•eIF4G complex [19]. The translation is also stimulated by mTOR via the phosphorylation of the serine-threonine kinase ribosomal protein S6 kinase 1 (S6K1 or p70S6K1) [20]. When amino acids are available, insulin regulates mTOR signaling through activation of upstream kinases such as Protein Kinase B, which phosphorylates serine 2448 of mTOR. Specifically, leucine is known to act with insulin to stimulate mTOR signaling proteins including ribosomal protein 6 (rpS6) and eIF4E [21].

Chromium (Cr) has an important effect on improving insulin action and increasing the metabolism of nutrients including carbohydrates, lipids, proteins, and nucleic acids by activation of enzymes involved in linked pathways including glucose transporters (GLUTs), insulin receptor substrate-1 (IRS-1), and fatty acid synthase (FAS) [22–25]. Because amylopectin may also lead to a rapid increase in blood glucose and insulin concentrations [26], an amylopectin/chromium complex (ACr) has been studied for its ability to augment exercise-induced MPS. In a previous clinical study, it was shown that intake of ACr with WP directly before exercise doubles the rate of MPS compared to the same dose of WP alone [17]. To date, however, no studies have reported on the detailed mechanisms of action (e.g. mTOR pathway) of BCAAs combined with ACr. Thus, the aim of the present study was to investigate the effects BCAAs combined with ACr on MPS, insulin concentration, and the mTOR signaling pathway.

## Material and methods

### Animal and study design

Twenty-four male Wistar rats (8 weeks old) were reared at 22 ± 2 °C in a 12/12-h light/dark cycle and fed with a basal diet and water ad libitum. All study procedures were approved by the Ethical Committee of the Firat University Animal Experiments (Elazig, Turkey). The rats were randomly divided into three groups as follows (*n* = 8 per group): (I) Exercise group, (II) Exercise plus BCAAs (0.465 g/kg BW, a 6 g human equivalent dose (HED)), and (III) Exercise plus BCAAs (0.465 g/kg BW, a 6 g HED) and ACr (0.155 g/kg BW, a 2 g HED). BCAAs contained L-leucine, L-isoleucine, and L-valine in a 2:1:1 ratio. ACr (Velositol®) was provided by Nutrition 21, NY, USA. ACr, a combination of chromium (500 mcg) from chromium picolinate and chromium histidinate and amylopectin from waxy maize (895 mg/g), has attained Generally Recognized as Safe (GRAS) status after an extensive and rigorous review of the scientific dossier and safety data. Doses of BCAAs and ACr were converted by allometric scaling, which takes into account differences in body surface area to calculate equivalent doses for rats by the following calculation, assuming an 80 kg human:

$$ \mathrm{RD}=\mathrm{HD}\left(\mathrm{g}\right)/80\;\mathrm{kg}\ast 6.2 $$

where RD is the rat dosage of BCAAs or ACr, HD is the human dosage of BCAAs or ACr, and 6.2 is the conversion factor to convert a human dosage to a rat dosage. Study product was dissolved in water and administered via oral gavage immediately following exercise.

All rats went through a 10-day treadmill acclimation schedule that gradually increased in speed and duration up to 26 m/min for 15 min on a motor-driven treadmill. The treadmill was supplied with an electric shock grid on the rear barrier to provide exercise motivation to the rats. On the day of the single dose trial, animals exercised on a treadmill at 26 m/min for 2 h and then were administered BCAAs, BCAAs plus ACr, or water immediately after the exercise, according to the assigned group.

Approximately 1 h after administration of study product and exercise, animals were injected with a deuterium-labeled phenylalanine bolus dose (250 mg/kg body weight, 25 g/L) to determine the fractional rate of protein synthesis (FSR). Ten minutes later, animals were sacrificed by decapitation and hind limbs were quickly removed and immersed in an ice-water mixture [21]. After removal from cooled hind limbs, muscles were frozen in liquid N_2,_ and stored at − 80 °C, for 3–6 h, for analysis of MPS. In addition, blood samples were collected 12 h after gavage and following decapitation in centrifuge tubes and were centrifuged at 3000×g for 10 min. The right gastrocnemius muscle was rapidly excised and stored at − 80 °C for Western blot analysis. Study personnel who were responsible for data collection and analysis were blinded to the study treatments.

### Laboratory analyses

#### FSR analysis

The frozen muscle was pulverized in liquid nitrogen and protein was precipitated with cold (4 °C) perchloric acid (30 g/L, 1 mL per 50 mg tissue) [21]. After centrifugation, the supernatants were collected, and the pellets were further washed with distilled water and hydrolyzed with hydrochloric acid. Protein synthesis was measured in muscles via the incorporation of injected [^2^H_5_] phenylalanine into muscle proteins [27]. Determination of [^2^H_5_] phenylalanine enrichment in plasma samples and in hydrolyzed muscle protein samples have been described previously [27]. GC-MS of the t-butyldimethylsilyl derivative under electron impact and selective ion recording was used to measure the enrichment of [^2^H_5_] phenylalanine in the muscle free amino acid pool [28]. The fractional rates of protein synthesis, FSR, were determined from the rate of incorporation of L-2 H5-phenylalanine into total mixed muscle protein as described previously [21, 27]. The amount of time for L-2 H5-phenylalanine incorporation was measured as the time from injection of the metabolic tracer until tissue cooling. FSR, defined as the percentage of tissue protein renewed each day, was calculated using the following formula:

$$ \mathrm{FSR}=\left(\mathrm{Eb}\times 100\right)/\left(\mathrm{Ea}\ \mathrm{x}\ \mathrm{t}\right) $$

where t is the time interval between injection and the snap freezing of muscle expressed in days, and Eb and Ea are the enrichment of [^2^H_5_] phenylalanine in hydrolyzed tissue protein and in muscle free amino acids, respectively.

#### Amino acid analysis

In order to measure free amino acids, serum samples were deproteinized with acidified methanol (8.4 ml 0.1 M HCl/100 ml methanol). The mixture was left for 20 min at 4 °C and centrifuged for 10 min at 15, 400×g. Amino acid levels were measured with an LC system containing a Varian Modular Analytical HPLC Systems quaternary pump with a degasser and photodiode array detector. Samples were injected at 5 μL to an autosampler with a thermostatic column section on an ACE–5 C18 column (5 μ, 250 mm; 4.6 mm) at 40 °C. Data analyses were done with ChemStation. A mobile phase was a linear gradient from 2 to 22% (0.1% formic acid and 0.01% hexafluorobutyric acid in acetonitrile) over 10 min, from 22 to 80% (0.1% formic acid and 0.01% hexafluorobutyric acid in acetonitrile) over 10 min and then maintained at 80% (0.1% formic acid and 0.01% hexafluorobutyric acid in acetonitrile) for an additional 6 min; the flow rate was 0.8 mL/min. Peak area integration was implemented by Analyst version 1.5 Intelli Quan quantitation software (Applied Biosystems, Foster City, CA, USA) [28]. Sample measurements were repeated in triplicate. The limits of detection (LODs) and limits of quantification (LOQs) of the tests ranged between 0.20–1.0 mg/kg and 1.0–5.0 mg/kg, respectively, depending on the amino acid under consideration. In addition, recoveries in the range of 80–103% were detected for samples at three concentrations (low, mid, and high) covering the working range of the method. The precision of the method, in terms of repeatability and reproducibility, was below 10% (percentage relative to standard deviation) for the amino acids analyzed.

#### Insulin analysis

Serum insulin concentration was analyzed with Rat Insulin Kits (Linco Research Inc., St. Charles, MO, USA) via ELISA device (Elx-800, Bio-Tek Instruments Inc., Vermont, USA). The assay sensitivity was 0.22 ng/mL and inter- and intra- assay constants were 4.7 and 5.8%.

### Western blot analysis

Levels of mTOR, S6K1, and 4E-BP1 in the muscle were analyzed by Western blot method [29]. For this purpose, 50 μg of protein was transferred to a nitrocellulose membrane after electrophoresis (Schleicher and Schuell Inc., Keene, NH, USA). The phosphorylated forms of antibodies against mTOR, S6K1 and 4E-BP1 proteins (Abcam, Cambridge, UK) were diluted in a concentration of (1:1000) in a PBS buffer which contains 0.05% Tween®-20. The loading of proteins was controlled by a monoclonal mouse antibody versus β-actin (A5316; Sigma). The bands (Supp. Fig. 1) were viewed with Image J, an image analysis system (National Institute of Health, Bethesda, USA).

### Statistical analyses

The data were statistically analyzed by one-way ANOVA using the SPSS statistical program (IBM, SPPS Version 21). Differences between the groups were achieved by Tukey post hoc test for the multiple comparisons that were performed in the analyses, and *P* < 0.05 was considered statistically significant. Data were reported as mean and standard deviations.

## Results

### Fractional rate of protein synthesis (FSR)

**Fig. 1:**
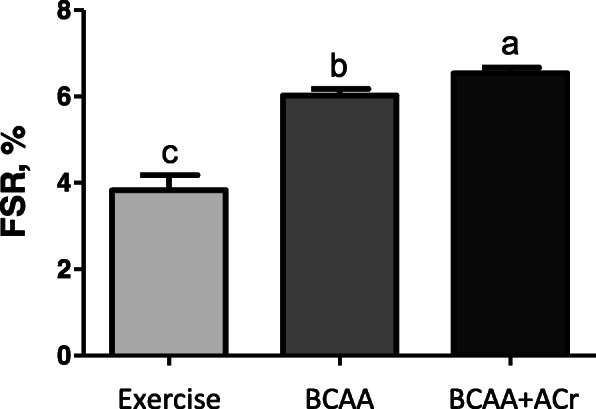
The effect of supplementation with an amylopectin/chromium complex and branched-chain amino acids on the fractional rate of protein synthesis (FSR). Values represent the means and standard errors of three different analyses. Significant between-group differences are represented with different letters (*p* < 0.05)

There was an increase in FSR, measured in percent/day, in the BCAAs and BCAAs plus ACr groups compared to the exercise control group (*p* < 0.05). However, the BCAAs plus ACr group increased MPS by 71% over the exercise control group, compared to a 57% increase in the BCAAs alone group (*p* < 0.05) (Fig. 1).

### Serum free amino acid levels

Because mTOR signaling is dependent on adequate free amino acid levels, all essential and non-essential amino acids levels were analyzed from serum samples after exercise and ingestion of study product. BCAAs and BCAAs plus ACr produced significantly higher serum BCAA levels (leucine, isoleucine, and valine) compared to exercise control rats (*p* < 0.0001; Table 1). Moreover**,** the addition of ACr to BCAAs increased leucine levels by 87%, isoleucine levels by 70%, and valine levels by 18% compared to BCAAs alone, over exercise controls (*p* < 0.05). Serum alanine, arginine, asparagine, histidine, lysine, and methionine levels were significantly increased in the BCAA plus ACr group compared to the BCAAs and exercise alone groups (*p* < 0.05). Serum glutamine and glutamate increased in the BCAAs plus ACr group compared to exercise controls (*p* < 0.05). There were no differences in serum aspartate (*p* = 0.866), citrulline (*p* = 0.662), glycine (*p* = 0.671), proline (*p* = 0.3921), serine (*p* = 0.617), threonine (*p* = 0.111), tryptophan (*p* = 0.239), and tyrosine (*p* = 0.482) levels among the groups (Table 1).

**Table 1:** The effect of an amylopectin/chromium complex and branched-chain amino acids on serum amino acids levels

Amino acid	Groups		
	Exercise	BCAAs	BCAAs + ACr
Alanine	356.55 ± 9.75^c^	408.27 ± 11.18^b^	466.47 ± 9.76^a^
Arginine	59.20 ± 2.73^b^	65.77 ± 1.76^b^	78.02 ± 3.37^a^
Asparagine	40.42 ± 1.94^c^	57.73 ± 2.25^b^	66.88 ± 2.29^a^
Aspartate	32.61 ± 1.65	34.07 ± 2.69	33.99 ± 2.00
Citrulline	78.53 ± 4.27	75.40 ± 2.33	80.21 ± 4.29
Glutamate	114.81 ± 3.33^b^	125.67 ± 3.73^ab^	149.18 ± 10.05^a^
Glutamine	315.28 ± 3.53^b^	339.60 ± 5.98^a^	363.21 ± 2.54^a^
Glycine	266.95 ± 16.65	279.58 ± 20.31	286.77 ± 6.82
Histidine	97.67 ± 3.26^c^	115.20 ± 2.99^b^	127.37 ± 2.88^a^
Isoleucine	61.25 ± 2.84^c^	194.11 ± 6.70^b^	236.86 ± 7.59^a^
Leucine	90.75 ± 5.57^c^	382.74 ± 15.81^b^	461.55 ± 15.89^a^
Lysine	373.00 ± 9.96^b^	464.87 ± 14.82^b^	507.56 ± 13.46^a^
Methionine	40.19 ± 2.11^c^	56.81 ± 2.88^b^	70.51 ± 4.10^a^
Proline	70.69 ± 4.44	79.87 ± 6.50	82.90 ± 7.72
Serine	301.93 ± 15.42	322.57 ± 21.71	323.67 ± 13.92
Threonine	260.86 ± 11.10	297.06 ± 11.62	293.15 ± 14.33
Tryptophan	45.62 ± 1.29	43.83 ± 1.83	50.37 ± 4.10
Tyrosine	41.24 ± 2.45	37.95 ± 1.63	42.20 ± 3.28
Valine	212.50 ± 5.08^c^	234.61 ± 5.64^b^	272.66 ± 6.99^a^

The data are presented as mean and standard error. ^a-c^ Mean values within the same row with different superscripts are statistically different (*p* < 0.05)

### Insulin levels

**Fig. 2:**
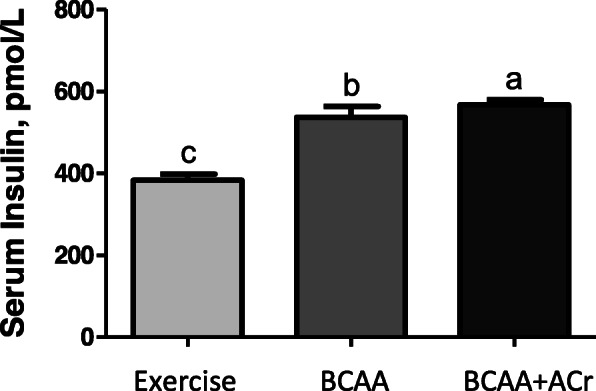
The effect of supplementation with an amylopectin/chromium complex and branched-chain amino acids on serum insulin levels. Values represent the means and standard errors of three different analyses. Significant between-group differences are represented with different letters (*p* < 0.05)

Because mTOR signaling is also dependent on adequate insulin levels, serum insulin levels were analyzed after exercise and ingestion of study product. Serum insulin levels increased by 48% in the BCAAs plus ACr group over the exercise control group, compared to 40% in the BCAAs alone group (*p* < 0.05). Examining all groups, the highest serum insulin levels were observed in the BCAAs plus ACr group (Fig. 2).

### mTOR, S6K1 and 4E-BP1 levels

**Fig. 3:**
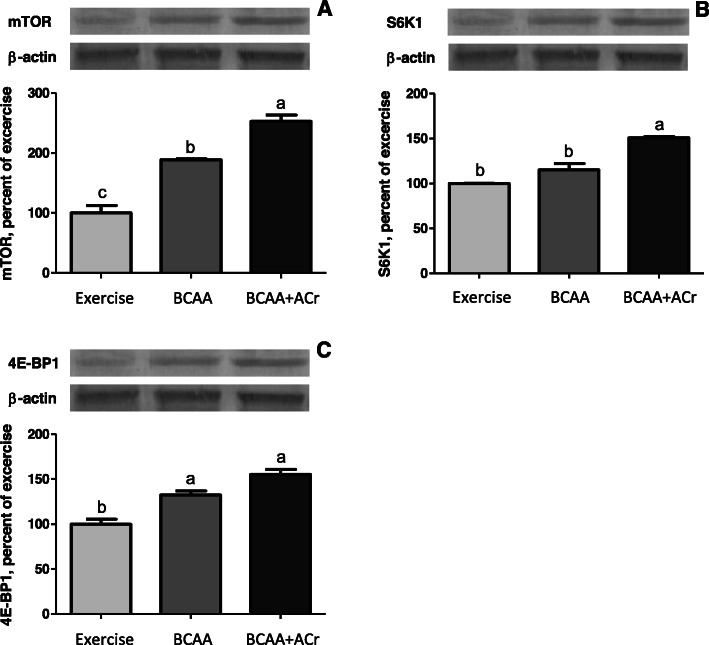
The effect of supplementation with an amylopectin/chromium complex and branched-chain amino acids on protein levels of the mammalian target of rapamycin (mTOR), ribosomal protein S6 kinase beta-1 (S6K1) and eukaryotic translation initiation factor 4E-binding protein 1 (4E-BP1). mTOR (**a**), S6K1 (**b**) and 4E-BP1 (**c**) in rat tissues, detected by Western blot analysis. Data are the percent of the exercise (set to 100%). Values represent the means and standard errors of three different analyses. Actin expression was used to ensure equal protein loading. Significant between-group differences are represented with different letters (*p* < 0.05)

To evaluate the effect of combining ACr with BCAAs on the MPS signaling cascade, the phosphorylation of 4E-BP1, S6K1, and mTOR were analyzed after exercise and ingestion of study product. Compared to the exercise control group, there was an increase in mTOR phosphorylation at the Ser2448 level in both experimental groups (*p* < 0.05). There was an increase in mTOR phosphorylation of 153% for BCAAs plus ACr and 89% for BCAAs, over the exercise control group (*p* < 0.05) (Fig. 3a). Compared to the exercise control group, there was only a substantial increase in S6K1 phosphorylation at the Thr389 level for the BCAAs plus ACr group (*p* < 0.05). There was an increase in S6K1 phosphorylation of 51% for the BCAAs plus ACr group (*p* < 0.05) and 15% for the BCAAs group, over the exercise control group (Fig. 3b). Compared to the exercise control group, there was an increase in 4E-BP1 phosphorylation levels for both experimental groups (*p* < 0.05). Although there was no significant difference between groups, there was an increase in 4E-BP1 phosphorylation of 55% for BCAAs plus ACr and 32% for BCAAs alone, over the exercise control group (Fig. 3c).

There were positive correlations between FSR and insulin concentration (*r* = 0.948; *p* < 0.001), mTOR, (*r* = 0.928; *p* < 0.001), S6K1 (*r* = 0.789; *p* < 0.05), and 4E-BP1 (*r* = 0.887; *p* < 0.01) levels. Moreover, insulin concentration was positively correlated with levels of mTOR, (*r* = 0.923; *p* < 0.001), S6K1 (*r* = 0.814; *p* < 0.01), and 4E-BP1 (*r* = 0.953; *p* < 0.001) (Table 2).

**Table 2:** Pearson’s correlation coefficients (r) correlations among FSR, insulin, mTOR, pS6K1, and 4E-BP1

	Insulin	mTOR	pS6K1	4E-BP1
FSR	0.948^a^	0.928^a^	0.789^c^	0.887^b^
Insulin		0.923^a^	0.814^b^	0.953^a^
mTOR			0.897^b^	0.900^b^
pS6K1				0.895^b^

^a^Correlation is significant at the 0.001 level

^b^Correlation is significant at the 0.01 level

^c^Correlation is significant at the 0.05 level

## Discussion

Previous studies have shown that supplementation with BCAAs or leucine alone results in significant rises in MPS rates with a concomitant increase in the phosphorylation of downstream targets of mTOR signaling in exercised rats [30, 31]. Increases in MPS due to EAAs are associated with an increase in signaling activity in the mTOR / p70S6K pathway [32]. In the present study, results showed that ACr combined with BCAAs significantly enhanced MPS, serum insulin levels, and amino acid levels compared to BCAAs alone and exercise alone. While this is the first study to examine ACr in combination with BCAAs, prior research has investigated the effects of ACr on MPS when combined with whey protein. A clinical model demonstrated that ingestion of ACr + 6 g of whey protein prior to a leg extension exercise protocol resulted in significantly greater MPS compared to 6 g of whey protein alone [17]. Moreover, in a preclinical study of similar design to the present study, researchers found that ACr significantly increased MPS when combined with increasing doses of whey protein compared to whey protein alone. When comparing these findings to the present data, it is evident that the combination of BCAAs and ACr increased FSR by a similar amount as 20 g (HED) of whey protein alone [33]. Although it has been suggested that the use of BCAAs alone for MPS is unwarranted [34], the results from the present study support previous clinical research showing that BCAA supplementation promotes MPS [35].

Results from the present study also demonstrated that ACr combined with BCAAs improved phosphorylation of mTOR, S6K1, and 4E-BP1 compared to BCAAs alone and exercise alone. These effects may be due to the ACr complex providing an extra advantageous biochemical situation through which proteins such as mTOR can be synthesized. Moreover, the phosphorylation states of mTOR, S6K1 and 4E-BP1 were greatly associated with insulin concentrations in this study. Based on these current results and earlier studies, it can be speculated that the elevated MPS stimulated via the ACr complex is mediated by the activation of the mTOR signaling pathway. The mTOR pathway, a significant controlling factor for MPS, stimulates the kinase activity of the complex and leads to the phosphorylation of 4E-BP1 and p70S6K, which are two enzymes that also modulate protein synthesis at the level of mRNA translation initiation [36, 37]. The capacity of 4E-BP1 to release eIF4E from the 4E-BP1eIF4E complex to induce mRNA translation is mainly dependent on the 4E-BP1 hyperphosphorylation level. Several nutrients, such as amino acids and carbohydrates, stimulate protein synthesis via the activation of the mTOR/p70S6K and e4E-BP1 pathway [38]. Previous studies showed that EAAs plus carbohydrates promoted better MPS post-exercise than control and that this MPS was linked to more robust phosphorylation of mTOR and p70S6k [39, 40]. Additionally, leucine and insulin have been stated to stimulate phosphorylation of mTOR at Ser2448 [41, 42]. Similar to our results, Wang et al. [4] reported that MPS was significantly increased by whey protein plus carbohydrates compared with placebo, and approached significance compared to whey protein alone. They also reported that whey protein plus carbohydrates produced superior phosphorylation of mTOR and p70S6K compared with the sedentary and placebo groups. In addition, Yoshida et al. [7] reported that treadmill exercise elevated the phosphorylation of p70S6 kinase in the muscle of low protein diet-fed chronic kidney disease (CKD) rats. They also reported that the BCAAs of the CKD rats restored the phosphorylation of p70S6 kinase to the same level detected in the sham group, however the rise in MPS and muscle mass was marginal. Morrison et al. [39] reported that supplementing rats with a solution comprised of either carbohydrates, protein, or carbohydrates plus protein immediately following exercise rapidly improved the phosphorylation of mTOR, 4E-BP1 and p70S6K compared with exercise control rats. In a study investigating protein and carbohydrate (50% sucrose plus 50% maltodextrose) supplementation, both carbohydrate plus soy protein and carbohydrate plus whey protein improved formation of the mRNA cap-binding complex eIF4F and stimulated phosphorylation of the translational repressor, 4E-BP1, S6K1, and mTOR compared with carbohydrates alone [31]. In another study, however, it was reported that neither hindlimb suspension nor chromium treatment changed the protein levels of myostatin, phospho-Forkhead box O-, or mTOR [43].

The purported ability of chromium to favorably enhance insulin metabolism is possibly a mechanism by which ACr enhances the mTOR mediated MPS pathway during muscle recovery when combined with BCAAs [22, 23, 44]. Previous studies have shown that chromium enhances GLUT-4 translocation by increasing insulin receptor activation, which results in improved insulin sensitivity and glucose uptake [23]. For instance, studies done by our groups and others have reported that chromium picolinate (CrPic)/chromium histidinate (CrHis) may enhance carbohydrate and lipid metabolism by regulation of glucose transporters, PPAR-γ and p-IRS-1 expression, and other insulin metabolism aspects [22–24, 45]. It has also been reported that supplemental CrHis/CrPic elevates liver GLUT-2 levels, as well as muscle Nrf2 and GLUT-4 levels, and reduces muscle NF-κB levels, with CrHis being superior to CrPic [46]. Chromium’s mechanism of action is crucial for MPS because when insulin binds to muscle cells, it stimulates the transportation of EAAs into the muscle cells. It has been established that insulin has a stimulating effect on MPS when acceptable EAA precursors are present and works to reduce muscle protein degradation when EAA levels are decreased [16, 17]. The transportation of EAAs into muscle cells is important for the activation of certain mTOR signaling proteins, such as S6K1 and 4E-BP1, that are responsible for regulating muscular growth [31]. For instance, one study found that when orally administering leucine or a carbohydrate meal to rats after exercise, MPS only increased in the leucine group, demonstrating the importance of adequate levels of amino acids for MPS stimulation. These increases in MPS were also correlated with changes in the phosphorylation of S6K1 and 4E-BP1, demonstrating that leucine stimulates MPS through the mTOR signaling pathway [30]. The connection between amino acid concentration and insulin-induced mTOR signaling is supported by clinical research showing that an increase in plasma amino acid levels by amino acid infusion increases insulin-stimulated mTORC1/S6K1 activity [47]. Additionally, leucine and insulin have been stated to stimulate phosphorylation of mTOR at Ser2448 [41]. Therefore, the mTOR signaling pathway is mediated by the presence and action of both amino acids and insulin, and lack of either may result in reduced MPS. By combining BCAAs with ACr, a factor that is known to positively impact insulin metabolism, the mTOR signaling pathway is provided with two of its critical components.

The anabolic benefits of post-exercise ACr and BCAA supplementation may be attributed to the fact that physical exercise normally causes an amino acid imbalance by promoting proteolysis relative to protein synthesis in skeletal muscle, leading to a decrease in plasma BCAA levels [48]. Interestingly, results from the current experiment showed that ACr increased serum concentrations of BCAAs and other amino acids despite their known utilization in muscle during exercise. Because animals were fasted overnight, de novo synthesis of certain amino acids may have occurred to compensate for a shortage of non-essential amino acids in the gut, as suggested by Wolfe et al. [34]. Furthermore, treatment with BCAAs, along with ACr, may have led to selective utilization of amino acids by muscle tissue, sparing glucogenic amino acids and S-containing amino acids.

While further clinical research is needed, results of the present experiment, along with data from previous preclinical and clinical studies, may be of interest to athletic and fitness communities who are interested in supplementing with a dietary supplement (ACr; Velositol®) in combination with a source of protein or amino acids to stimulate muscle anabolism and in turn, result in greater muscular outcomes from exercise. These results could also be of high relevance to aging populations who may have a harder time gaining and maintaining muscle, as aging can result in resistance to the anabolic effects of amino acids and negative alterations in glucose metabolism [17, 49]. Chromium has been clinically shown to improve cholesterol and glucose levels in non-diabetic and diabetic subjects, as well as result in body fat loss and increased muscle gains in resistance trained men [23, 50]. Consequently, ACr may enable older populations to gain muscle mass, reduce the risk of injury, and support overall health.

To further explore the effect of ACr on augmenting MPS, clinical outcomes studies should be carried out in the future to examine the effect of ACr and BCAAs on muscular growth and strength. Furthermore, because the present study was conducted as a single dose experiment, it would be of interest to examine the effect of consuming BCAAs and ACr after exercise over a longer period. Moreover, because the present study only examined the effect of the addition of ACr to a 6 g HED of BCAAs, it would be of interest to examine how ACr would affect MPS when added to various doses of BCAAs. Because MPS is strongly reliant on EAA levels, it is possible that adding ACr to increasing doses of BCAAs or EAAs will magnify the rates of MPS seen with intake of ACr plus a 6 g HED of BCAAs.

## Conclusions

This study showed that the addition of an amylopectin/chromium complex (ACr; Velositol®) to BCAAs improved MPS by 71% over the exercise controls, compared to a 57% increase in the BCAAs alone group. These results provide evidence that the MPS boosting effect of ACr seen in the prior clinical study [17] using whey protein may also occur when using BCAAs. The results also suggest that BCAAs combined with ACr may be more effective in improving the mTOR pathway compared to BCAAs alone in exercised rats. To further explore this mechanism, this study should be replicated in a clinical model. Overall, the results from this preclinical study support the use of an amylopectin/chromium complex as a bioactive sports nutrition ingredient to support MPS with BCAAs.

## Supplementary information

**Additional file 1 **: **Figure S1**. Western Blot bands for mammalian target of rapamycin (mTOR), ribosomal protein S6 kinase beta-1 (S6K1), eukaryotic translation initiation factor 4E-binding protein 1 (4E-BP1), and β-actin levels.

## Data Availability

The data and materials for this manuscript are not scheduled to be made publicly available due to the proprietary nature of the investigated materials. Contractually, the data is owned by Nutrition 21, LLC, not any of the authors.

## Abbreviations

ACr: Amylopectin/chromium complex
BCAAs: Branched-chain amino acids
BW: Body weight
EAAs: Essential amino acids
eIF4E: Eukaryotic translation initiation factor 4E
FSR: Fractional rate of protein synthesis
MPS: Muscle protein synthesis
mTOR: mammalian target of rapamycin
rpS6: ribosomal protein S6
S6K1: Ribosomal protein S6 kinase beta-1
WP: Whey protein
4E-BP1: Eukaryotic translation initiation factor 4E-binding protein 1

## References

[1] Philp A, Hargreaves M, Baar K (2012). More than a store: regulatory roles for glycogen in skeletal muscle adaptation to exercise. Am J Physiol Endocrinol Metab.

[2] McGlory C, van Vliet S, Stokes T, Mittendorfer B, Phillips SM (2019). The impact of exercise and nutrition on the regulation of skeletal muscle mass. J Physiol.

[3] Ivy JL, Ding Z, Hwang H, Cialdella-Kam LC, Morrison PJ (2008). Post exercise carbohydrate-protein supplementation: phosphorylation of muscle proteins involved in glycogen synthesis and protein translation. Amino Acids.

[4] Wang W, Ding Z, Solares GJ, Choi SM, Wang B, Yoon A, Farrar RP, Ivy JL (2017). Co-ingestion of carbohydrate and whey protein increases fasted rates of muscle protein synthesis immediately after resistance exercise in rats. PLoS One.

[5] Boutry C, El-Kadi SW, Suryawan A, Wheatley SM, Orellana RA, Kimball SR (2013). Leucine pulses enhance skeletal muscle protein synthesis during continuous feeding in neonatal pigs. Am J Physiol Endocrinol Metab.

[6] Falavigna G, Alves de Araújo J, Rogero MM, Pires IS, Pedrosa RG, Martins E, Alves de Castro I, Tirapegui J (2012). Effects of diets supplemented with branched-chain amino acids on the performance and fatigue mechanisms of rats submitted to prolonged physical exercise. Nutrients.

[7] Yoshida T, Kakizawa S, Totsuka Y, Sugimoto M, Miura S, Kumagai H (2017). Effect of endurance training and branched-chain amino acids on the signaling for muscle protein synthesis in CKD model rats fed a low-protein diet. Am J Physiol Renal Physiol.

[8] Shimomura Y, Murakami T, Nakai N, Nagasaki M, Harris RA (2004). Exercise promotes BCAA catabolism: effects of BCAA supplementation on skeletal muscle during exercise. J Nutr.

[9] Tajiri K, Shimizu Y (2018). Branched-chain amino acids in liver diseases. Transl Gastroenterol Hepatol.

[10] Biolo G, Fleming RY, Maggi SP, Wolfe RR (1995). Transmembrane transport and intracellular kinetics of amino acids in human skeletal muscle. Am J Physiol Endocrinol Metab.

[11] Gonçalves NG, Cavaletti SH, Pasqualucci CA, Arruda Martins M, Lin CJ (2017). Fructose ingestion impairs expression of genes involved in skeletal muscle's adaptive response to aerobic exercise. Genes Nutr.

[12] Muñoz VR, Gaspar RC, Kuga GK, da Rocha AL, Crisol BM, Botezelli JD, Baptista IL, Mekary RA, da Silva ASR, Cintra DE, de Moura LP, Ropelle ER, Pauli JR (2018). Exercise increases rho-kinase activity and insulin signaling in skeletal muscle. J Cell Physiol.

[13] Churchward-Venne TA, Burd NA, Mitchell CJ (2012). Supplementation of a suboptimal protein dose with leucine or essential amino acids: effects on myofibrillar protein synthesis at rest and following resistance exercise in men. J Physiol.

[14] Kanda Atsushi, Nakayama Kyosuke, Sanbongi Chiaki, Nagata Masashi, Ikegami Shuji, Itoh Hiroyuki (2016). Effects of Whey, Caseinate, or Milk Protein Ingestion on Muscle Protein Synthesis after Exercise. Nutrients.

[15] Borsheim E, Cree MG, Tipton KD, Elliott TA, Aarsland A, Wolfe RR (2004). Effect of carbohydrate intake on net muscle protein synthesis during recovery from resistance exercise. J Appl Physiol.

[16] Wolfe RR (2000). Effects of insulin on muscle tissue. Curr Opin Clin Nutr MetabCare.

[17] Ziegenfuss TN, Lopez HL, Kedia A, Habowski SM, Sandrock JE, Raub B, Kerksick CM, Ferrando AA (2017). Effects of an amylopectin and chromium complex on the anabolic response to a suboptimal dose of whey protein. JISSN.

[18] Schiaffino S, Dyar KA, Ciciliot S, Blaauw B, Sandri M (2013). Mechanisms regulating skeletal muscle growth and atrophy. FEBS J.

[19] Kimball SR, Shantz LM, Horetsky RL, Jefferson LS (1999). Leucine regulates translation of specific mRNAs in L6 myoblasts through mTOR-mediated changes in availability of eIF4E and phosphorylation of ribosomal protein S6. J Biol Chem.

[20] Wullschleger S, Loewith R, Hall MN (2006). TOR signaling in growth and metabolism. Cell..

[21] Norton LE, Wilson GJ, Layman DK, Moulton CJ, Garlick PJ (2012). Leucine content of dietary proteins is a determinant of postprandial skeletal muscle protein synthesis in adult rats. Nutr Metabol.

[22] Wang H, Kruszewski A, Brautigan DL (2005). Cellular chromium enhances activation of insulin receptor kinase. Biochemistry..

[23] Cefalu WT, Wang ZQ, Zhang XH (2002). Oral chromium picolinate improves carbohydrate and lipid metabolism and enhances skeletal muscle glut-4 translocation in obese, hyperinsulinemic (JCR-LA corpulent) rats. J Nutr.

[24] Sahin K, Tuzcu M, Orhan C, Sahin N, Kucuk O, Ozercan IH, Juturu V, Komorowski JR (2013). Anti-diabetic activity of chromium picolinate and biotin in rats with type 2 diabetes induced by high-fat diet and streptozotocin. Br J Nutr.

[25] Chen G, Gao Z, Chu W, Cao Z, Li C, Zhao H (2018). Effects of chromium Picolinate on fat deposition, activity and genetic expression of lipid metabolism-related enzymes in 21 day old Ross broilers. Asian-Australas J Anim Sci.

[26] Regmi PR, Matte JJ, Van Kempen TATG, Zijlstra RT (2010). (2010). Starch chemistry affects kinetics of glucose absorption and insulin response in swine. Livest Sci.

[27] Bark T, McNurlan M, Lang C, Garlick PJ (1998). Increased protein synthesis after acute IGF-I or insulin infusion is localized to muscle in mice. Am J Phys.

[28] Takach E, O'Shea T, Liu H (2014). 2014. High-throughput quantitation of amino acids in rat and mouse biological matrices using stable isotope labeling and UPLC-MS/MS analysis. J Chromatogr B Analyt Technol Biomed Life Sci.

[29] Sahin K, Pala R, Tuzcu M, Ozdemir O, Orhan C, Sahin N, Juturu V (2016). Curcumin prevents muscle damage by regulating NF-κB and Nrf2 pathways and improves performance: an in vivo model. J Inflamm Res.

[30] Anthony JC, Anthony TG, Kimball SR, Vary TC, Jefferson LS (2000). Orally administered leucine stimulates protein synthesis in skeletal muscle of postabsorptive rats in association with increased eIF4F formation. J Nutr.

[31] Anthony TG, McDaniel BJ, Knoll P, Bunpo P, Paul GL, McNurlan MA (2007). Feeding meals containing soy or whey protein after exercise stimulates protein synthesis and translation initiation in the skeletal muscle of male rats. Nutr..

[32] Rennie MJ, Bohé J, Smith K, Wackerhage H, Greenhaff P (2006). Branched-chain amino acids as fuels and anabolic signals in human muscle. J Nutr.

[33] Komorowski J, Perez Ojalvo S, Sahin N, Tastan H, Sahin K (2017). The effect of the addition of an amylopectin/chromium complex to increasing doses of whey protein on muscle protein synthesis in rats. JISSN..

[34] Wolfe RR (2017). Branched-chain amino acids and muscle protein synthesis in humans: myth or reality?. JISSN..

[35] Jackman SR, Witard OC, Philp A, Wallis GA, Baar K, Tipton KD (2017). Branched-chain amino acid ingestion stimulates muscle Myofibrillar protein synthesis following resistance exercise in humans. Front Physiol.

[36] Bouitbir J, Sanvee GM, Panajatovic MV, Singh F, Gingras AC KS, Gygi SP (2019). Mechanisms of statin-associated skeletal muscle-associated symptoms. Pharmacol Res.

[37] Ogasawara Riki, Jensen Thomas E., Goodman Craig A., Hornberger Troy A. (2019). Resistance Exercise-Induced Hypertrophy. Exercise and Sport Sciences Reviews.

[38] Kim D, Sarbassov D, Ali S, King J, Latek R, Erdjument-Bromage H, Tempst P, Sabatini D (2002). mTOR interacts with raptor to form a nutrientsensitive complex that signals to the cell growth machinery. Cell..

[39] Morrison PJ, Hara D, Ding Z, Ivy JL (2008). Adding protein to a carbohydrate supplement provided after endurance exercise enhances 4E-BP1 and RPS6 signaling in skeletal muscle. J Appl Physiol.

[40] Dreyer HC, Drummond MJ, Pennings B, Fujita S, Glynn EL, Chinkes DL (2008). Leucine-enriched essential amino acid and carbohydrate ingestion following resistance exercise enhances mTOR signaling and protein synthesis in human muscle. Am J Physiol Endocrinol Metab.

[41] Proud C (2004). Role of mTOR signaling in the control of translation initiation and elongation by nutrients. Curr Top Microbiol Immunol.

[42] Smith G, Yoshino J, Stromsdorfer KL, Klein SJ, Magkos F, Reeds DN, Klein S, Mittendorfer B (2015). Protein ingestion induces muscle insulin resistance independent of Leucine-mediated mTOR activation. Diabetes..

[43] Dong F, Hua Y, Zhao P, Ren J, Du M, Sreejayan N (2009). Chromium supplement inhibits skeletal muscle atrophy in hindlimb-suspended mice. J Nutr Biochem.

[44] Sahin K, Onderci M, Tuzcu M, Ustundag B, Cikim G, Ozercan IH, Sriramoju V, Juturu V, Komorowski JR (2007). Effect of chromium on carbohydrate and lipid metabolism in a rat model of type 2 diabetes mellitus: the fat-fed, streptozotocin-treated rat. Metabol Clin Exp.

[45] Orhan C, Sahin N, Tuzcu Z, Komorowski JR, Sahin K (2017). Combined oral supplementation of chromium picolinate, docosahexaenoic acid, and boron enhances neuroprotection in rats fed a high-fat diet. Turk J Med Sci.

[46] Sahin N, Hayirli A, Orhan C, Tuzcu M, Akdemir F, Komorowski JR, Sahin K (2017). Effects of the supplemental chromium form on performance and oxidative stress in broilers exposed to heat stress. Poult Sci.

[47] Yoon M (2016). The emerging role of branched-chain amino acids in insulin resistance and metabolism. Nutrients..

[48] Ji LL, Miller RH, Nagle FJ, Lardy HA, Stratman FW (1987). Amino acid metabolism during exercise in trained rats: the potential role of carnitine in the metabolic fate of branched-chain amino acids. Metabolism.

[49] Kalyani RR, Egan JM (2013). Diabetes and altered glucose metabolism with aging. Endocrinol Metab Clin N Am.

[50] Kaats GR, Blum K, Pullin D, Keith SC, Wood R (1998). A randomized, double-masked, placebo-controlled study of the effects of chromium picolinate supplementation on body composition: a replication and extension of a previous study. Curr Ther Res.

