# Supplementary material for: The addition of an amylopectin/chromium complex to branched-chain amino acids enhances muscle protein synthesis in rat skeletal muscle
Source: J Int Soc Sports Nutr. 2020 May 27;17:26. doi: 10.1186/s12970-020-00355-8 (PMC7251890; doi:10.1186/s12970-020-00355-8)

Supplementary Figure 1. Western Blot bands for mammalian target of rapamycin (mTOR), ribosomal protein S6 kinase beta-1 (S6K1), eukaryotic translation initiation factor 4E-binding protein 1 (4E-BP1), and β-actin levels.

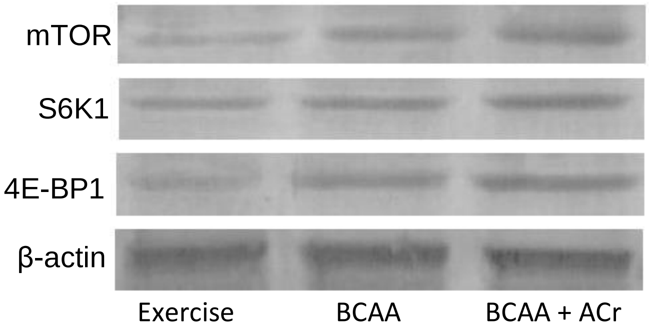

Supplement: Supplementary file 1 — Additional file 1 : Figure S1. Western Blot bands for mammalian target of rapamycin (mTOR), ribosomal protein S6 kinase beta-1 (S6K1), eukaryotic translation initiation factor 4E-binding protein 1 (4E-BP1), and β-actin levels. [file 12970_2020_355_MOESM1_ESM.pdf]
